# Deep carbon cycle constrained by carbonate solubility

**DOI:** 10.1038/s41467-021-24533-7

**Published:** 2021-07-14

**Authors:** Stefan Farsang, Marion Louvel, Chaoshuai Zhao, Mohamed Mezouar, Angelika D. Rosa, Remo N. Widmer, Xiaolei Feng, Jin Liu, Simon A. T. Redfern

**Affiliations:** 1 Department of Earth Sciences, University of Cambridge, Downing Street, Cambridge, CB2 3EQ UK; 2 Institut für Mineralogie, WWU Münster, Münster, 48149 Germany; 3 Center for High Pressure Science and Technology Advanced Research (HPSTAR), Beijing, 100094 China; 4 European Synchrotron Radiation Facility, 71 Avenue des Martyrs, Grenoble, 38000 France; 5 Empa, Swiss Federal Laboratories for Materials Science and Technology, Laboratory for Mechanics of Materials and Nanostructures, Feuerwerkerstrasse 39, Thun, 3602 Switzerland; 6 Asian School of the Environment, Nanyang Technological University, 50 Nanyang Avenue, Singapore, 639798 Singapore

**Keywords:** Geochemistry, Mineralogy

## Abstract

Earth’s deep carbon cycle affects atmospheric CO_2_, climate, and habitability. Owing to the extreme solubility of CaCO_3_, aqueous fluids released from the subducting slab could extract all carbon from the slab. However, recycling efficiency is estimated at only around 40%. Data from carbonate inclusions, petrology, and Mg isotope systematics indicate Ca^2+^ in carbonates is replaced by Mg^2+^ and other cations during subduction. Here we determined the solubility of dolomite [CaMg(CO_3_)_2_] and rhodochrosite (MnCO_3_), and put an upper limit on that of magnesite (MgCO_3_) under subduction zone conditions. Solubility decreases at least two orders of magnitude as carbonates become Mg-rich. This decreased solubility, coupled with heterogeneity of carbon and water subduction, may explain discrepancies in carbon recycling estimates. Over a range of slab settings, we find aqueous dissolution responsible for mobilizing 10 to 92% of slab carbon. Globally, aqueous fluids mobilise ${35}_{-17}^{+20}$% (${27}_{-13}^{+16}$ Mt/yr) of subducted carbon from subducting slabs.

## Abstract (web summary)

Carbonate mineral aqueous solubility decreases as carbonates become more Mg-rich during subduction. Coupled with regional variations in amounts of carbon and water subducted, this explains discrepancies in estimates of carbon recycling, suggesting that only around a third returns to the surface.

## Introduction

**Fig. 1:**
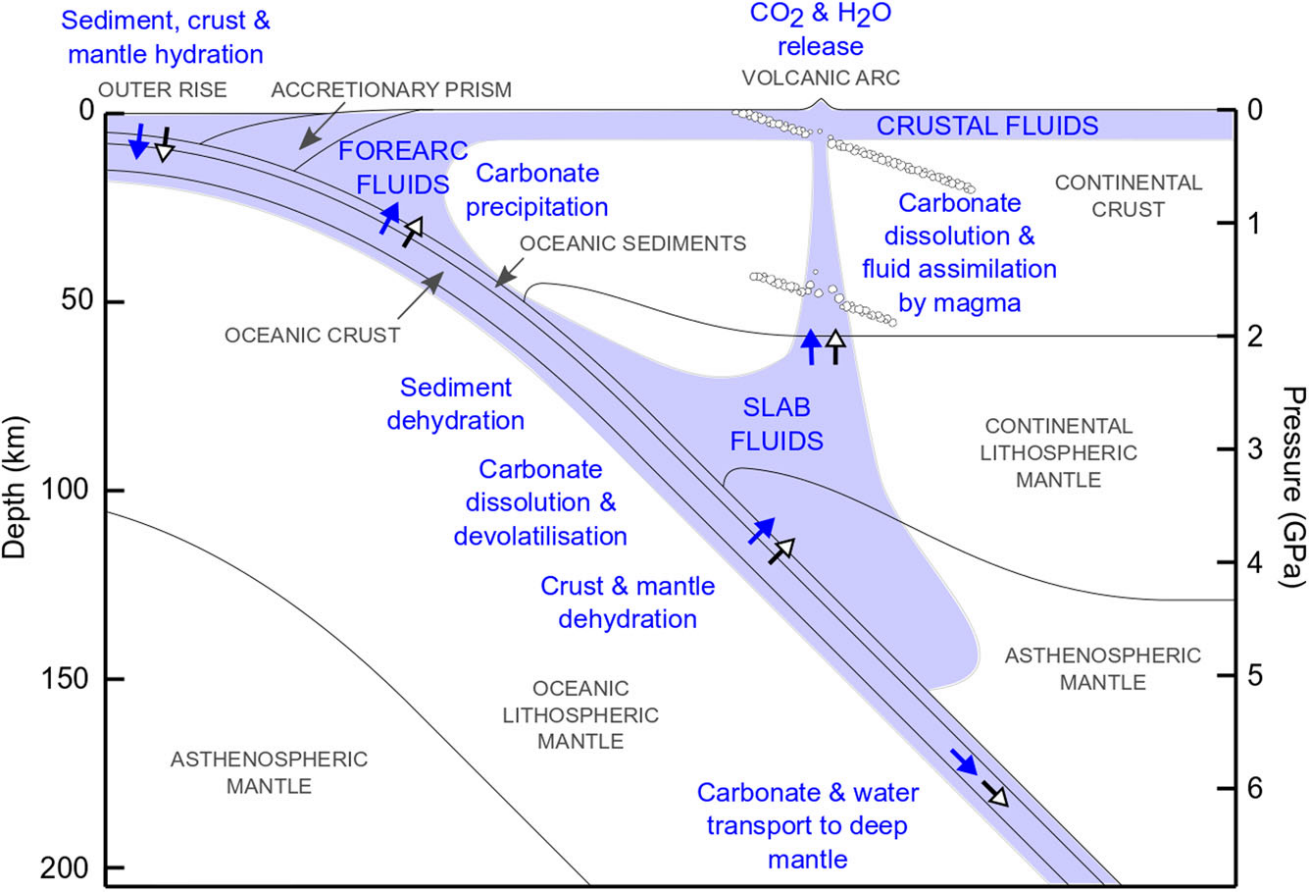
Fluid–carbonate mineral interactions in the deep carbon cycle. White headed black arrows indicate carbonate flux and blue arrows water flux. Blue shaded areas indicate water-rich regions. The melting of carbonated igneous oceanic crust is not shown as it starts at depths of 300 km^22^. The image is to scale, apart from the thickness of oceanic sediments that has been exaggerated.

Two modes of tectonic carbon cycling are associated with subduction zones^1^. The first is the shallow accretionary carbon cycle that includes the accretion of shelf, oceanic island, and minor seafloor sedimentary carbon to continents, the incorporation and transport of this carbon by ascending fluids and magmas, followed by its release to the atmosphere or oceans. The second is the deeper carbon cycle that includes the subduction of carbon, the devolatilization, aqueous dissolution, or melting of carbon-bearing phases, the transport of liberated CO_2_ to the mantle wedge and volcanic arc followed by active or passive degassing. Finally, carbon may be transferred to the deep mantle in high-pressure carbon-bearing phases. The extent to which each of these processes operates is contentious (Fig. 1).

Each subduction zone has unique compositional and physical characteristics reflected in water and carbon flux heterogeneity^2–5^. Each year, at least 1 Gt of water^2^ bound in hydrous minerals and 78 Mt of carbon^3,4^ stored in carbonate minerals or as reduced organic carbon gets transported to deep Earth via the subduction of three major lithologies: oceanic sediments, igneous crust, and lithospheric mantle. Oceanic sediments rich in carbonate shells and the organic remains of marine and terrestrial organisms hydrate at the time of their deposition on the ocean floor. Sedimentary carbon dominates the carbon input into the mantle with 80% of the carbon stored in calcite and aragonite shells and 20% as organic carbon^3^. The upper oceanic crust undergoes hydrothermal aqueous alteration on its journey from mid-ocean ridges to trenches. Carbonate minerals in hydrothermally altered igneous oceanic crust are predominantly calcite and aragonite^5,6^, but magnesite, dolomite^7^, siderite (FeCO_3_), and ankerite [Ca(Fe,Mg)(CO_3_)_2_] may be also present^8^. Lower crust and lithospheric mantle hydration and carbonation may take place at linear spreading centers due to extensional faulting^9^ or at the outer rise, as a consequence of extensional bend faulting of the subducting slab^10,11^. Mantle peridotite then alters to carbonated serpentinites, also known as ophicarbonates, which may contain calcite, dolomite, and/or magnesite^12^.

Water from the subducting slab gets released either by early-stage sediment compaction or by gradual dehydration reactions that occur in the sediments, igneous crust, and lithospheric mantle (e.g., deserpentinisation^13^) down to as deep as the transition zone—lower mantle boundary^14^. The degree and depth of dehydration strongly depend on composition, local pressure, and temperature^2^ and is enhanced as the subducting slab approaches the high-temperature mantle wedge. Slab-derived fluids exhibit a wide range of compositions, with typical solute contents of <15 wt% in forearc fluids and <30 wt% in subarc fluids^15^, although concentrations as high as >50 wt% may occur^16^. In hot subduction zones, water release to the mantle wedge can also happen through ascending hydrous melts produced by the melting of subducted sediments^17^ and igneous crust (e.g., adakitic magmas^18^ and carbonatitic melts^19^).

Processes removing carbon from the subducting slab include mechanical removal, and the devolatilisation, melting, and dissolution of carbonate minerals. Experimental studies^20^ and thermodynamic modeling^21^ indicate that only very little metamorphic devolatilization of carbonates takes place under subduction zone conditions. Furthermore, it has been shown that geotherms characteristic of subducting slabs intersect the melting curve of carbonated igneous oceanic crust at depths of 300–700 km, hence significantly reducing the stability region of carbonate minerals^22^. Therefore, carbonate dissolution in slab-derived fluids may become the dominant mechanism for carbon removal up to these depths. Carbonate dissolution has been reported in many different subduction environments. Near surface, authigenic carbonate is, for instance, deposited by serpentinite diapirs venting fluids^23^. With increasing depth (10–50 km), fluid–rock interaction results in the formation of fully carbonated peridotites composed of magnesite and quartz or dolomite and quartz, also known as listvenites^24^. Even deeper, high-pressure rocks preserve evidence for the carbonate dissolution. Ultrahigh-pressure rocks from the Italian western Alps or marbles from the Cycladic subduction complex on the Syros and Tinos islands, Greece, for instance, contain solid and fluid inclusions enriched in bicarbonate and carbonate ions and different carbonate minerals (calcite, magnesite, dolomite, and less common rhodochrosite)^25,26^. Carbonate O and C isotope systematics serve as additional evidence for carbonate dissolution accompanied by silicate precipitation^26^. Calcite, intermediate composition calcium–magnesium carbonate (Ca_0.75_Mg_0.25_CO_3_) (and the coexistence of each with ice VII), and dolomite inclusions in diamonds suggest the interaction region of water and carbonate minerals extends to at least transition zone depths^14,27^.

Experiments demonstrating the extreme solubility of calcium carbonate (CaCO_3_) in high *P*–*T* fluids above 1 GPa and 700 °C (refs. ^28–31^) further suggest the critical role of slab-derived fluids in recycling carbon to the surface, with an efficiency that could reach almost 100% (ref. ^31^). However, CO_2_ degassing at volcanic arcs represents only a fraction of subducted carbon, the other main carbon outputs being degassing of mid-ocean ridge basalts and diffuse degassing associated with intraplate volcanism^5,32^. Although carbon input and output fluxes from the mantle are very similar^5^, carbon recycling estimates at volcanic arcs may be complicated by a number of processes, including crustal assimilation of carbonate host rocks^33,34^ and slab carbon sequestration within the crust by carbonate deposition^35^. Whereas calcite and aragonite are the dominant carbonate minerals at the Earth’s surface, observations of carbonates in deep fluid and solid inclusions^14,25,27^ indicate that these may not be the sole phases contributing to the deep carbon cycle. Experimental petrological work^36–38^ indeed suggests that, with increasing depth, dolomite (~2 GPa) and magnesite (~4 GPa) become the most stable carbonate phases. Their predominance above ~2 GPa is further supported by Mg isotope systematics that point toward the coexistence of Mg and Ca-rich phases in deep subduction zone settings^39^. More exotic carbonate species may also occur in the deep Earth. For instance, the presence of rhodochrosite in ultrahigh-pressure rocks is associated with the subduction of deep-sea ferromanganese nodules^25^. The current lack of information on the high *P*–*T* solubility of these minerals is striking and obscures our understanding of the deep carbon cycle.

Here, we determine the solubility of dolomite [CaMg(CO_3_)_2_] and rhodochrosite (MnCO_3_), and put an upper limit on that of magnesite (MgCO_3_) in high *P*–*T* H_2_O–NaCl fluids. We combine these with available data on calcite/aragonite^28–30^, smithsonite (ZnCO_3_)^40^, and strontianite (SrCO_3_)^41^, to define novel constraints on carbon recycling by subduction zone fluids. We find carbonate solubility decreases by more than two orders of magnitude on transformation from Ca- to Mg-rich. This decreased solubility, coupled with heterogeneity of carbon and water subduction, may explain discrepancies in carbon recycling estimates. Depending on the nature of subducting slab, we find aqueous dissolution is responsible for mobilizing no more than 92%, and potentially as little as 10%, of slab carbon content. We estimate that ${35}_{-17}^{+20}$% of the global subducted carbon gets mobilized by aqueous fluids, corresponding to the annual recycling of ${27}_{-13}^{+16}$ Mt of carbon from the subducting slabs.

## Results and discussion

### Solubility of carbonates in high *P*–*T* subduction zone fluids

**Fig. 2:**
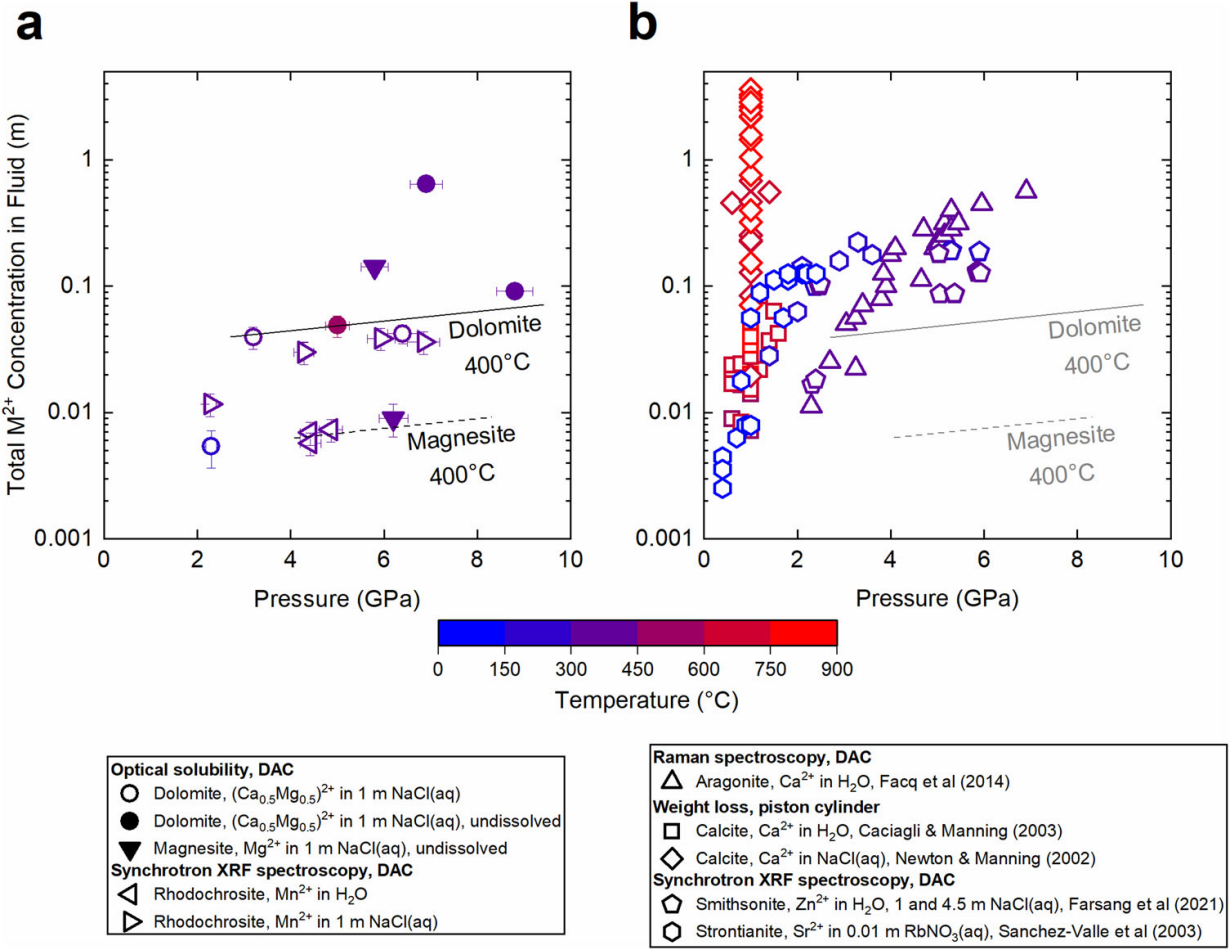
Experimental determinations of carbonate mineral solubilities. Solubility is reported as a function of pressure and temperature in water or aqueous solutions of different ionic strength: **a** present data, **b** literature data. Only studies at pressures of 1 GPa or more are included. Full symbols indicate upper limits for solubility. The solid line represents a solubility line for dolomite at 400 °C in 1 m NaCl aqueous solution. Since no magnesite crystals dissolved, the dashed line represents an upper solubility limit for magnesite at 400 °C. All solubility lines are guides to the eye. Error bars shown for our data represent estimated standard deviation. Note the log scale.

The aqueous solubility of dolomite and magnesite was investigated by optical solubility experiments and the aqueous solubility of rhodochrosite by synchrotron X-ray fluorescence spectroscopy (see “Methods”). Our solubility data are presented along with published results on calcite/aragonite, smithsonite, and strontianite in Fig. 2 and Table 1. In general, carbonate solubility increases with *P*, *T*, and may reach extreme values just below melting conditions^28,29,40,42,43^. Carbonate solubility is also higher in saline fluids compared to pure water. The high *P*–*T* solubility of carbonates follows the order strontianite > calcite/aragonite > dolomite, rhodochrosite, smithsonite ≫ magnesite, (Fig. 2), with a striking (more than two orders of magnitude) difference in calcite/aragonite versus magnesite solubility as indicated by earlier ab initio molecular dynamics calculations^44^.

**Table 1:** Carbonate solubility data.

Mineral and Fluid	Pressure (GPa)	Temperature (°C)	Concentration (m)
Optical solubility experiments			
Dolomite, CaMg(CO_3_)_2_ in 1 m NaCl(aq)	6.9 (3)	400 (2)	0.32 (2)^a^
	3.2 (2)	412 (2)	0.020 (4)
	2.3 (1)	200 (2)	0.0027 (9)
	6.4 (3)	400 (2)	0.021 (4)
	8.8 (4)	400 (2)	0.046 (4)^a^
	5.0 (3)	500 (2)	0.024 (5)^a^
Magnesite, MgCO_3_ in 1 m NaCl(aq)	5.8 (3)	400 (2)	0.14 (2)^a^
	6.2 (3)	400 (2)	0.009 (3)^a^
Synchrotron X-ray fluorescence spectroscopy experiments			
Rhodochrosite, MnCO_3_ in H_2_O	4.4 (2)	300 (2)	0.007 (1)
	4.4 (2)	300 (2)	0.006 (1)
	4.9 (2)	300 (2)	0.007 (2)
Rhodochrosite, MnCO_3_ in 1 m NaCl(aq)	2.3 (1)	300 (2)	0.012 (2)
	4.3 (2)	300 (2)	0.030 (6)
	4.3 (2)	300 (2)	0.030 (6)
	5.9 (3)	300 (2)	0.039 (8)
	6.8 (3)	415 (2)	0.036 (7)

^a^Crystals that would represent these solubilities did not dissolve, hence these values represent maximum solubilities.

The observed solubility trends reflect competing effects of *P*, *T*, and salinity on the structure of supercritical aqueous fluid. The structural changes strongly affect the fluid’s most important physicochemical properties driving dissolution, including the relative dielectric permittivity (dielectric constant, *ε*_r_)^45^ and the level of self-ionization^46^. From ambient to our peak experimental *P*–*T* conditions, the *ε*_r_ of water is lowered by a factor of two^44^, while the ion product of water (*K*_w_) rises by ten orders of magnitude^47^.

In the isostructural (either orthorhombic or rhombohedral) carbonates, differences in physicochemical properties arise from the nature (e.g., size, mass, and electronic configuration) of the divalent metal cations (M^2+^), and the bonds between these and the O atoms of CO_3_^2-^ complex oxyanions. In naturally occurring calcite and aragonite group carbonates, the size of M^2+^ ranges from 0.69 Å for Ni to 1.47 Å for Ba. The Ca^2+^ ion is special, because it is both the smallest the orthorhombic aragonite structure and the largest the rhombohedral calcite structure can accommodate. The size of M^2+^ is reflected in O coordination and hence number of M^2+^–O bonds (nine in orthorhombic and six in rhombohedral phases). Typical electronic configurations include *p*^6^ closed-shells in alkaline earth metal ions, giving rise to strongly ionic M^2+^–O bonds, and partially or fully filled *d* orbitals in 3*d* transition metal ions, resulting in bonds with some covalent character. Due to the enhanced ionicity of high *P–T* fluids, carbonates with alkaline earth M^2+^ are expected to exhibit higher solubilities. However, these can still be orders of magnitude different, as seen for calcite and magnesite, most probably because of differences in ion size and anharmonicity^48^. The properties of M^2+^ also control speciation in liquids, which further affects solubility. While Ca^2+^ may form a range of hydrous, chlorinated, and carbonated complexes in high *P*–*T* Cl-rich fluids^49^, Zn^2+^ will almost exclusively form chlorinated species^40^.

### Implications for carbon recycling

Carbonate dissolution and release from the subducting slab is controlled by the interplay between carbonate phase stability and the availability of water. First, the highly soluble calcite and aragonite dissolve that have been carried to depth in porous sediments and in veins of igneous oceanic crust. Second, sedimentary dolomite and that formed by the reaction of CaCO_3_ and Mg-silicates in the igneous oceanic crust dissolve. Finally, the very low solubility magnesite, formed in the igneous oceanic crust and mantle, is most probably carried to subarc depths, as previously suggested^39^. The very high solubility of CaCO_3_ phases even at moderate pressures (~1 GPa) and high temperatures has important consequences for the shallow accretionary carbon cycle too. Almost half of subduction zones have accretionary prisms^50^. In these, fluid migration along fracture zones^51^ and the dissolution of accreted carbonate materials into these fluids may lead to significant shallow carbon recycling. However, other highly soluble minerals (e.g., gypsum, CaSO_4_·2H_2_O) may help saturate these aqueous fluids and slow down carbon recycling.

**Fig. 3:**
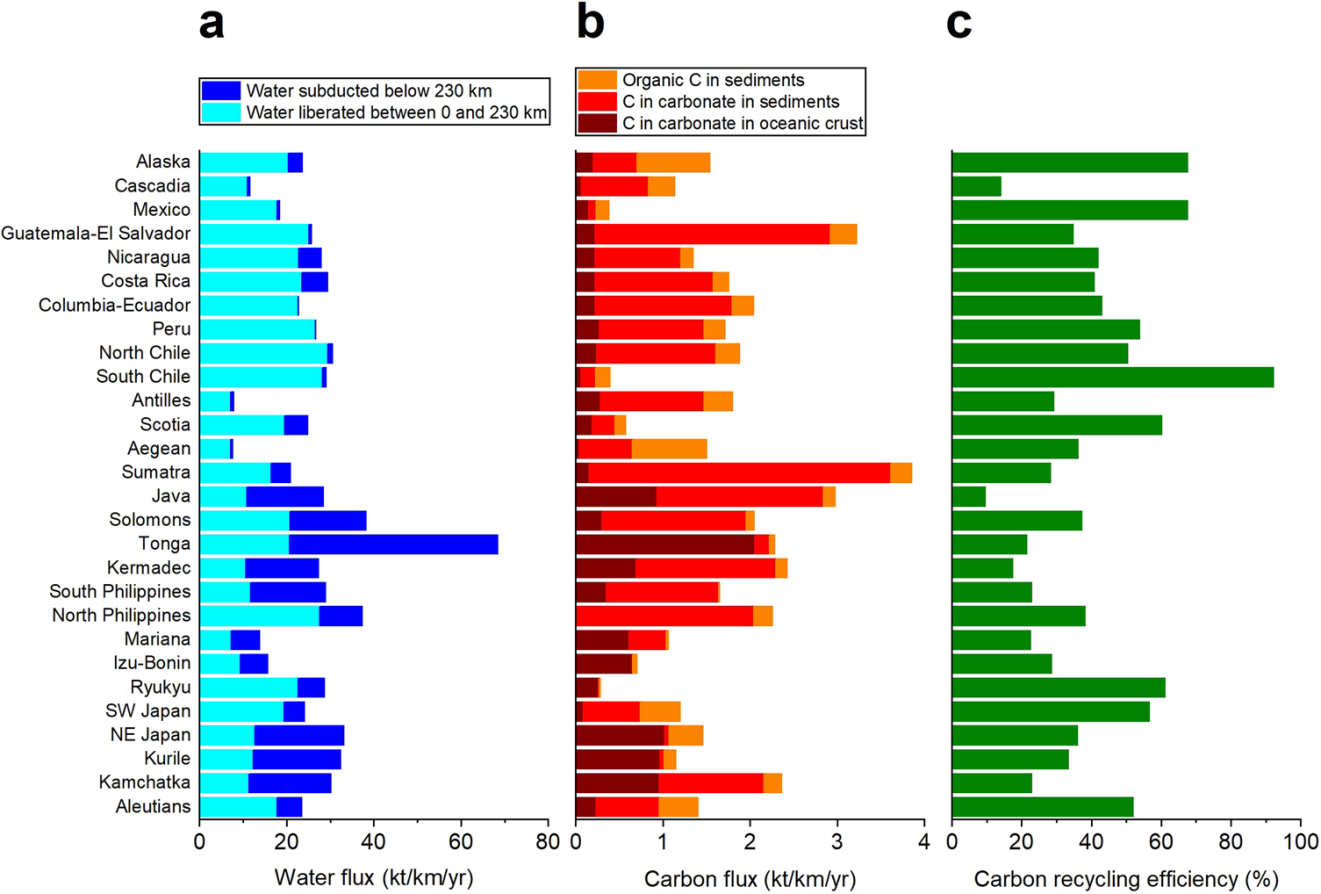
Heterogeneity of subduction zone compositions and processes. Differences in water^2^ (**a**) and carbon^3,4^ (**b**) flux among subduction zones and corresponding C recycling efficiency (**c**). The water flux estimates assume moderate mantle hydration^2^.

The amount of water fluxed out of the slab varies with slab lithology and *P–T* conditions (see Fig. 3a and Supplementary Data 1). The average subduction zone water flux is estimated to be ~1 Gt year^−1^, with 7% bound in sediment, 63% in igneous crust, and 30% in mantle, but is highly heterogeneous among subduction zones^2^. Based on high *P–T* experiments^52^, an estimated 32% is lost down to 100 km, 14% between 100 and 150 km, and 20% between 150 and 230 km, leaving up to 34% to be potentially transported to depths greater than 230 km (~7 GPa)^2^. Carbonate (and organic carbon) dissolution will also be affected by the heterogeneity of both sedimentary and crustal carbon flux^3,4^ (see Fig. 3b and Supplementary Data 1). Finally, water also catalyzes phase transitions. An example is the calcite–aragonite transition observed <100 km (~3 GPa)^30^. Taking into account estimated water flux, and effect of *P–T* on carbonate mineral solubility, we used experimental and extrapolated solubility values (see “Methods”) to calculate the amount of recycled carbon from individual subducting slabs (see Fig. 3c and Supplementary Data 2). The contrasting water, carbonate and organic carbon contents of the slabs, and their different water liberation depth profiles result in a wide range of carbon recycling efficiencies spanning from 10% for Java up to 92% for South Chile. In the case of moderately hydrated slabs with major carbon subduction (e.g., Sumatra, Java), only a small fraction of subducting carbon gets recycled. On the other hand, slabs with moderate water and minor carbon subduction (e.g., Mexico, South Chile) may lose most of their carbon content. Note that besides water content, the amount of liberated water and the depth profile of water liberation are equally important for effective carbon recycling. Although Java has less carbon and more water influx, its carbon recycling efficiency is much lower than that of Sumatra, given the larger proportion of liberated water in Sumatra. The amount of liberated water strongly depends on slab temperature. While in the coldest slabs (e.g., Tonga, Java, Kermadec) more than half of the water gets subducted below 230 km, in the hottest slabs (e.g., Guatemala-El Salvador, Cascadia, South Chile) the amount of water subducted below 230 km is negligible^2^ (Fig. 3a). High slab temperature therefore affects carbon recycling in at least two major ways: via increased carbonate solubility and by providing large amounts of solvent (i.e., water). Combining our carbon recycling calculations for individual slabs, we estimate that 35% of the global subducted carbon gets mobilized by aqueous fluids and is recycled from the subducting slabs. Finally, we acknowledge the potentially large errors associated with the extrapolated solubility values. We estimate that these span approximately one log unit range. Decreasing or increasing all solubility values by half a log unit in the two highest *P–T* intervals (see Supplementary Data 2) will result in global carbon recycling efficiencies of 18 and 55%, respectively. Based on published carbon influx estimates^3,4^, a ${35}_{-17}^{+20}$% recycling efficiency corresponds to ${27}_{-13}^{+16}$ Mt of dissolved carbon annually.

While our new constraints on carbon outflux from the slab to the mantle wedge are generally slightly lower than previous estimates, which range from <10% up to 100% (refs. ^11,52^), several studies reported similar global values around 40% (refs. ^21,35,53^). In contrast to Kelemen and Manning’s (2015) upper-bound estimate of “what goes down mostly comes up”, our calculations indicate that bulk slab and slab carbonate composition will significantly affect the efficiency of carbon recycling and that the role of high *P*–*T* phase transformation of calcite to dolomite, and ultimately magnesite on the recycling efficiency of carbon has been underestimated.

Our carbon recycling estimates can be compared to CO_2_ outputs for each arc setting, in order to find out how much of the carbon dissolved from each subducting slab ultimately gets recycled to the atmosphere. While the quantification of CO_2_ outputs is hindered by the difficulties in sampling volcanic emissions on remote or small volcanoes, a recent review^54^ suggests that much of CO_2_ outputs actually originate from passive degassing of strong emitters. Most of these strong emitters have been regularly monitored over the past 10–20 years and extensive datasets are hence available to constrain their CO_2_ outputs^54^, although data collected in such a short period may not be representative of geological timescales. We thus compared the CO_2_ emissions of these strong emitter volcanoes with our subducting slab-specific carbon recycling calculations (Supplementary Data 3) and found that while a couple of volcanic arcs (Nicaragua and Solomons) produce much more CO_2_ than suggested by calculated carbon dissolution, most volcanic arcs produce less CO_2_ than that potentially dissolved from the associated slabs. In case of the Nicaragua arc, carbon output from strong emitters also exceeds input by subduction. Apart from sampling bias, these differences may reflect one or more of the following: remobilization of crustal carbonate by ascending magmas, melting of the subducted lithologies and/or overlying mantle wedge, carbonate precipitation, temporal changes in subduction style, and the redox state of subducting slabs. For instance, much of the excess CO_2_ produced by the largest volcanic emitters (e.g., in Central America) can be ascribed to crustal carbonate from accreted limestone platforms^34^. The formation of metasedimentary diapirs^11^ and the melting of subducting igneous crust starting around 300 km (10 GPa, 1350 °C) in hot subduction zones, may as well significantly accelerate carbon recycling and result in higher CO_2_ outputs^22^. Compositional, redox and *P–T* changes along the major fluid pathways, including lithological interfaces and through the hot mantle wedge will likely result in a solubility drop, and associated precipitation or carbonation of the mantle wedge, thus hindering carbon recycling to the surface^35,55,56^. Furthermore, temporal changes in subduction style and rate of carbon subduction also have the potential to slow down the deep carbon cycle. In the Archean, subducting slabs were hotter^57^ and the enhanced solubility of carbonates with *T* likely resulted in a much shallower carbon cycling and increased CO_2_ concentrations in the atmosphere. The emergence of cold subduction, combined with the increasing subduction carbon flux over the past 80 Myr (ref. ^58^), likely contributed to the accumulation of large quantities of carbon in slabs, a deeper carbon cycle, and drop in atmospheric CO_2_ levels.

Finally, our carbonate solubility measurements were conducted assuming oxidizing conditions in saline aqueous solutions, in which only oxidized carbon species (e.g., HCO_3_^−^ and CO_3_^2−^) are present^30,49^. However, the redox state of individual subducting slabs and the composition of upper mantle aqueous fluids may differ widely. In aqueous fluids at pressures above ~2.0–3.0 GPa, a rich variety of aqueous carbon species with different oxidation states between −4.0 (e.g., CH_4_) and +4.0 (e.g., CO_2_) can coexist in thermodynamic equilibrium, and these aqueous fluids can then coexist with immiscible hydrocarbon fluids^59^. Field evidence from the Alps and Alpine Corsica indeed shows the existence of reduced, H_2_-rich fluids, resulting from the reaction of aqueous fluids with serpentinized peridotites^60,61^. Galvez et al.^60^ showed that slightly reduced fluids may react with carbonate minerals to become saturated in carbon leading to precipitation of large amounts of graphite. Ultra-reduced fluids reacting with carbonate minerals may trigger the generation of CH_4_-bearing^61^, immiscible aqueous (C–O–H) and hydrocarbon (C–H) fluids^59,60^, and the precipitation of graphite. The composition and redox state of subducting slab fluids may therefore affect the fate of subducting carbon. In a closed system, with respect to carbon, in the presence of slightly reducing fluid, graphite may be locked into a subducting slab and the retained carbon then act as precursor to diamond formation^60^. In an open system, with ultra-reduced fluids, carbon in these fluids may be fully transferred to shallower reservoirs, or again retained in the slab^61^. The effects of hydrocarbon immiscibility on the mobility and reactivity of deep carbon remain poorly constrained^59^. High-pressure–temperature carbonate solubility experiments exceeding the conditions of the present study and conducted in a range of fluids are therefore critically needed.

## Methods

### Starting materials

Naturally occurring carbonate minerals were used in all solubility experiments. The origin of these carbonates, their calculated chemical formulae from electron probe microanalysis, measured unit cell parameters from X-ray powder diffraction, and Raman spectra are reported in ref. ^62^. The fluids used were double distilled and deionized water (*ρ* = 18 MΩ cm) and a 1 m NaCl aqueous solution, prepared using high purity NaCl (>99.999%, Sigma Aldrich). Reference solutions with distinct Mn^2+^ concentrations for in situ synchrotron X-ray fluorescence spectroscopy quantitative measurements were prepared using MnCl_2_·4H_2_O. Due to the hygroscopic nature of this chemical, the precise Mn concentration of solutions was determined by inductively coupled plasma mass spectroscopy.

### Rhodochrosite solubility by in situ synchrotron X-ray fluorescence spectroscopy

Rhodochrosite solubility experiments in pure water and 1 m NaCl aqueous solution were carried out at the high-pressure beamline ID27 of the European Synchrotron Radiation Facility in Grenoble, France^63^ (Supplementary Fig. 1a). The operating conditions of the storage ring were 6 GeV and 180–200 mA in top-up mode.

All experiments were conducted using membrane-type diamond anvil cells^64^ equipped with type Ia single crystal diamond anvils with culet diameters of 500 μm. A partially perforated diamond anvil with a remaining thickness of ~150 μm facing the XRF detector was employed to minimize the absorption of the fluorescence signal from the diamond. The diamonds were cleaned before use in H_2_SO_4_ for several hours. Rhenium gaskets were employed, pre-indented from an initial thickness of 200–~80 μm, then laser drilled with a 250 μm diameter hole, and lined with a 25 μm gold layer to prevent Re dissolution in the high *P*–*T* fluid.

Rhodochrosite crystals of known volume (~40 × 40 × 40 µm^3^) were loaded in the sample chamber (~2.5 × 10^6^ μm^3^) together with the aqueous solution (Supplementary Fig. 1b). Pressure was remotely controlled and increased stepwise to the target pressure. During cold compression and heating, we used the diffraction signal of gold and the thermal equation of state reported by ref. ^65^ to determine the pressure. The incident X-ray beam energy for X-ray diffraction was 20 keV. Diffraction data were recorded using a 165 mm diameter MarCCD XRD detector positioned on the downstream side of the DAC (Supplementary Fig. 1a). The CCD detector was placed on the axis of the beam, ~240 mm from the sample. A CeO_2_ standard was used to calibrate the distance, detector tilt, and rotation parameter. The 2D diffraction images were integrated using Dioptas software^66^. Uncertainties in pressure determination are estimated to be 5% of the pressure values at all investigated temperatures.

The DAC was heated resistively using an external heating device developed at the European Synchrotron Radiation Facility. In this setup, the DAC and its heater were enclosed in a vacuum chamber equipped with Mylar windows that permit the transmission of X-rays. A high vacuum of up to 10^−6^ bars ensured a homogenous heating during the long duration of the experiments and prevents the oxidation of the diamonds and heater. The setup further allows for a fine and remote control of the pressure and temperature^67^. For the latter, a *K*-type thermocouple is positioned close to the heater. A second thermocouple was placed in contact with one of the diamonds to monitor the sample temperature. Uncertainties in temperature determination are estimated to be ±2 °C.

X-ray fluorescence measurements at the ID27 beamline were conducted in forward transmission geometry. A fixed-exit double crystal monochromator equipped with two silicon (111) crystals was used to tune the X-ray beam energy to 20.0 keV (Supplementary Fig. 1a). The incoming X-ray beam was focused to a spot size of 3 × 3 (*h* *×* *w*) µm^2^ using a pair of Kirkpatrick–Baez mirrors. The intensity of the beam before and after the sample was measured using an ion chamber and a PIN diode detector, operating in the current integration mode, respectively.

The emitted fluorescence signal from the sample was collected using a HITACHI Vortex Si drift diode detector with a 40 mm^2^ active area and a sensitive layer thickness of 1 mm, aligned at a permanent angle of 22.7°, with respect to the incoming X-ray beam. The detector was equipped with XOS polycapillary focusing optics, which allows the extraction of emitted fluorescence signal from a small sample area of 50 × 50 (*h* × *w*) µm^2^, significantly eliminating the Compton scattering and diffused scattering from the sample environment^68^. The polycapillary was positioned in 50 mm distance from the sample. The use of polycapillary increases drastically the signal-to-noise ratio at the expense of a reduced flux of emitted photons reaching the detector. The polycapillary half lens was repositioned onto the X-ray beam focus before every XRF acquisition, by scanning the XRF detector horizontally and vertically. For this operation, the X-ray beam was placed onto the Re gasket and the intensity of the Re Lα (8.7 keV) fluorescence line was monitored during the scan. A microscope mounted on the back of the DAC was used to visualize the sample and to reposition it in the X-ray focal plane for XRD detection with a centering procedure. The current setup ensured the acquisition of XRF signals coming only from Mn species dissolved in the fluid.

For each run, the sample was initially pressurized to ~2 GPa and then heated up to 300 °C (Supplementary Fig. 2). The pressure was then increased to ~6 GPa in pressure increments of ~1–2 GPa. Finally, the temperature was increased to 400 °C. Generally, two XRF spectra were collected at each high *P*–*T* step ~30 and ~60 min after reaching the target *P*–*T*, each for 200 s. Before XRF data acquisition, the incoming beam position was optimized from fine-stepped scans across the sample chamber, while monitoring the XRF signal intensity. The focus of the polycapillary optics was regularly repositioned on the beam focus using the XRF signal from the Re gasket. The intensity of the incoming beam (*I*_0_) was measured immediately before and after each sample XRF collection, in order to correct for intensity fluctuations of the incoming beam.

XRF spectra (Supplementary Fig. 3) were analyzed using PeakFit software. For each XRF spectrum, the X-ray photon background was subtracted and the area under the characteristic XRF bands was determined by least squares fitting to Voigt profiles. Once corrected for *I*_0_ fluctuations and density effects induced by increasing pressure and temperature, concentrations of Mn are proportional to the area of the Mn Kα (5.9 keV) fluorescence lines, determined from the peak fitting, and our fluorescence calibrations. The intensity of the Mn Kα fluorescence line was calibrated for the experimental setup using MnCl_2_·4H_2_O reference solutions, with distinct Mn concentrations. Calibration and solubility measurements were conducted using the same experimental geometry (i.e., same beam optical path, constant distance between the sample and fluorescence detector, and DAC centered on the X-ray focal point). The possibility of fluorescence from the Mn solid phase was ruled out by (1) placing the rhodochrosite crystal at the edge of the sample chamber, (2) scanning the XRF detector through the sample chamber in directions perpendicular to the incoming beam to find the position of crystal, and (3) positioning the X-ray beam at a point in the fluid at least 50 µm distant from both the crystal and gasket. The fluorescence signal of the fluid (i.e., areas away from the crystal) was uniform, confirming fluid homogeneity, as well as the absence of any contribution from the solid.

Typical uncertainties in the calculated Mn concentration at high *P*–*T* conditions are <30% of the measured values and include errors arising from the calibration, using reference solutions and peak fitting errors.

### Dolomite and magnesite optical solubility experiments

The low X-ray emission energies of Mg *K* edge (1.3 keV) hindered the accurate monitoring of magnesite and dolomite solubility by synchrotron X-ray fluorescence spectroscopy. Instead, optical solubility experiments based on crystal volume computation^69^ have been used to estimate the solubility of these carbonate minerals.

A type Heating-II diamond anvil cell (Beijing Scistar Technology) equipped with type Ia diamond anvils (Almax easyLab) with culet diameters of 500 μm were used in optical solubility experiments. T301 steel gaskets were employed, pre-indented from an initial thickness of 250 μm to ~35–70 μm, then laser drilled with a 200 μm diameter hole.

For each optical solubility experiment, a carbonate (either dolomite or magnesite) single crystal was loaded in the sample chamber (Supplementary Fig. 1c). The chamber was subsequently filled with a 1 m NaCl aqueous solution, which was prepared using high purity NaCl (>99%, Alfa Aesar).

Pressure was increased by tightening the screws of the DAC, and monitored using the pressure and temperature induced shifts of the T and L Raman bands of carbonate minerals^62^, after 5 min of response time required for the carbonate bands to reach their equilibrium value. Uncertainties in pressure determination are estimated to be 5% of the pressure values. Raman spectra for pressure determination were collected across the 100–1200 cm^−1^ spectral range using a Renishaw RM1000 Raman spectrometer of 250 mm focal length at the Center for High Pressure Science and Technology Advanced Research (HPSTAR) in Beijing, China. A holographic grating of 2400 g mm^−1^ enabled a spectral resolution of ~4 cm^−1^. The excitation line at 532.05 nm was produced by a Renishaw RL532C100 laser source focused on the sample using an Olympus SLMPLN 20× long working distance objective.

The DAC was externally heated via the Joule effect using a Pt wire wound around an Al_2_O_3_ ring, surrounding the diamond anvils of the cell. Temperature was increased using a DC power supply. A *K*-type thermocouple was placed in contact with one of the diamonds to monitor the sample temperature. Uncertainties in temperature determination are estimated to be ±2 °C.

Rhombohedral cleavage fragments were chosen for the solubility experiments. The initial morphology of the crystal was modeled, and its volume calculated by the Kristall2000 software (Schilling, 2008). The crystal fragment was gradually brought to higher pressure–temperature conditions until full dissolution or to pressure–temperature conditions limited by experimental setup (Supplementary Fig. 2). At each pressure–temperature step, the crystal was left for 60 min to allow mineral–fluid equilibration^41^. Full dissolution of the crystal enabled the quantification of minimum solubility at the given conditions, while no apparent or partial dissolution constrained maximum solubility. Where partial dissolution was observed, the experiment was run for an elongated time to make sure the crystal does not continue to dissolve.

### Carbon recycling calculations

The quantification of carbon dissolution from individual subducting slabs requires the following information: the amount of subducted water, the magnitude of water liberation as a function of depth, the amount and form of subducted carbon, changes in the distribution of carbon-bearing mineral phases as a function of depth, and the solubility of carbon-bearing mineral phases at the relevant pressure–temperature conditions.

Slab-specific water influx and outflux values have been estimated previously^2^ (see Supplementary Data 1). Liberated water was calculated for depth intervals of 0–100, 100–150, and 150–180 km, hence we adopted these ranges for our calculations.

Slab-specific estimates of sedimentary carbonate, sedimentary organic carbon, and igneous crustal carbonate can be found in ref. ^3^ and ref. ^4^, respectively (see Supplementary Data 1). In the carbonate of the subducting slabs, the following initial distribution of carbon was assumed: 100% in aragonite/calcite in sediments, and 60% in aragonite/calcite, 20% in dolomite, and 20% in magnesite in altered igneous oceanic crust. At 100 and 150 km depth, 50% of existing aragonite/calcite was assumed to transform to dolomite and 50% of dolomite to magnesite (e.g., due to Mg^2+^ ↔ Ca^2+^ cation exchange).

Carbon-bearing species were assumed to dissolve in a specific order, starting with the leaching of organic carbon and followed by the dissolution of carbonate minerals (see Supplementary Data 2). This assumption is supported by (1) the organic C-rich nature of subduction zone fluids^70^, and the isotopic signature of asthenospheric to transition zone diamonds that is consistent with carbonated igneous oceanic crust, rather than sediments rich in organic carbon, being the dominant source of their carbon^4,71^. Even if some of the organic carbon does not dissolve and eventually graphitizes as the slab experiences higher pressures and temperatures with depth, the available water will dissolve calcite/aragonite instead. Since in most subduction zones there is only enough water for the dissolution of some organic carbon and calcite/aragonite, as long as the solubility of organic carbon and calcite/aragonite in subduction zone fluids are of a similar magnitude, the amount of recycled carbon should not be affected significantly. Of the carbonate minerals, calcite/aragonite was assumed to dissolve first, followed by dolomite and finally, magnesite. We acknowledge that some of the carbonate phases may dissolve simultaneously and we justify our simplification by the large differences in the solubility of these species.

Solubility values for dolomite and magnesite in 1 m NaCl(aq) at 400 °C (i.e., corresponding to the “shallow” dissolution of carbonates from 0 to 100 km depth) were taken from this work, while aqueous solubilities for calcite/aragonite^30^ were doubled to account for the effect of salinity. For the deeper transfer (100–150 and 150–230 km), available trends for calcite/aragonite were extrapolated^28–30^ (Table 2): the pressure effect was estimated from the experiments of ref. ^30^ conducted at 300, 350, and 400 °C, respectively up to ~7 GPa and the temperature effect from the experiments of ref. ^29^ conducted at 0.6, 0.8, and 1 GPa, respectively up to 800 °C. Relative solubility changes of dolomite and magnesite were assumed to follow those of calcite/aragonite. The errors associated with the extrapolated solubility values at 100–150 and 150–230 km are estimated to span a log unit range. Organic carbon was assumed to leach out at magnitude corresponding to calcite/aragonite dissolution. The lack of experimental data on the solubility of carbon-bearing phases at few GPa and >400 °C is one of the main limitations of our calculations. The uniform temperature at different depth ranges is a key simplification addressing the heterogeneity of subduction zone temperature profiles and very high-temperature gradients at the top of the subducting slabs, where most carbonates concentrate^72^. Due to the limitations and simplifications outlined above, our calculations should be treated as first approximation.

**Table 2:** Assumed average carbonate solubilities in 1 m NaCl aqueous solution as a function of depth.

Mineral	0–100 km (400 °C)	100–150 km (800 °C)	150–230 km (1000 °C)
Calcite/aragonite	0.2	6	12
Dolomite	0.02	0.6	1.2
Magnesite	0.005	0.15	0.3

Average temperatures refer to the top sections of slabs^72^, where carbonate minerals concentrate. All solubility values are in m.

## Supplementary information

Supplementary Information

Description of Additional Supplementary Files

sup data 1

sup data 2

sup data 3

## Data Availability

All relevant data are available from the authors on request and/or are included with the manuscript (in the form of data tables or data within figures). Source data are provided with this paper.

## Source data

Source Data
