# Supplementary material for: Deep carbon cycle constrained by carbonate solubility
Source: Nat Commun. 2021 Jul 14;12:4311. doi: 10.1038/s41467-021-24533-7 (PMC8280166; doi:10.1038/s41467-021-24533-7)
Supplement: Supplementary file 1 — Supplementary Information [file 41467_2021_24533_MOESM1_ESM.pdf]

Deep carbon cycle constrained by carbonate solubility

Stefan Farsang[1*], Marion Louvel[2], Chaoshuai Zhao[3], Mohamed Mezouar[4], Angelika D. Rosa[4], Remo N. Widmer[5], Xiaolei Feng[1,3], Jin Liu[3] and Simon A. T. Redfern[6#]

[1]Department of Earth Sciences, University of Cambridge, Downing Street, Cambridge, CB2 3EQ, UK ([*]sf571@cam.ac.uk)

[2]Institut für Mineralogie, WWU Münster, 48149 Münster, Germany

[3]Center for High Pressure Science and Technology Advanced Research (HPSTAR), 100094 Beijing, China

[4]European Synchrotron Radiation Facility, 71 Avenue des Martyrs, 38000 Grenoble, France

[5]Empa, Swiss Federal Laboratories for Materials Science and Technology, Laboratory for Mechanics of Materials and Nanostructures, Feuerwerkerstrasse 39, Thun, 3602, Switzerland

[6]Asian School of the Environment, Nanyang Technological University, 50 Nanyang Avenue, Singapore, 639798, Singapore ([#]simon.redfern@ntu.edu.sg)

Supplementary information

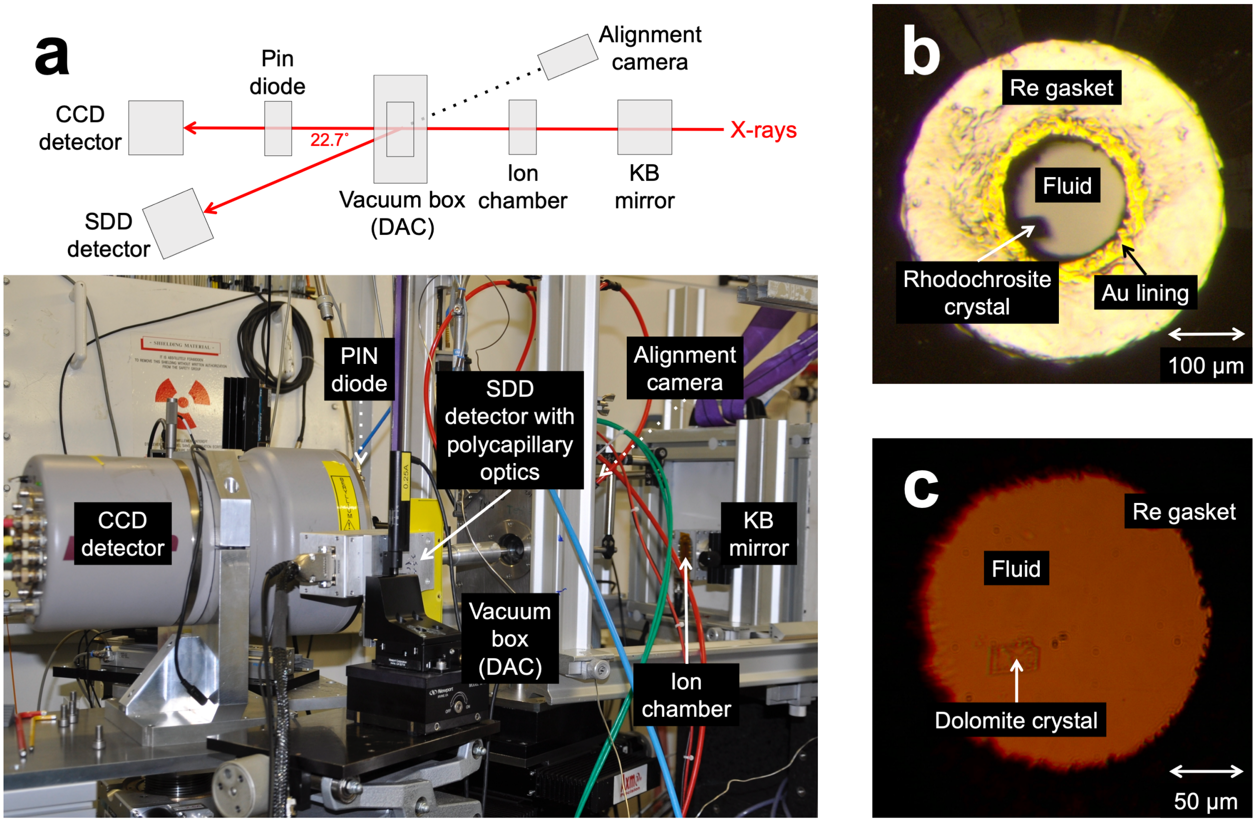

Supplementary Figure 1. a) Schematic diagram and image of the experimental setup at the ID27 beamline of ESRF. b) A DAC loaded for synchrotron XRF spectroscopy experiments. c) A DAC loaded for optical solubility experiments.

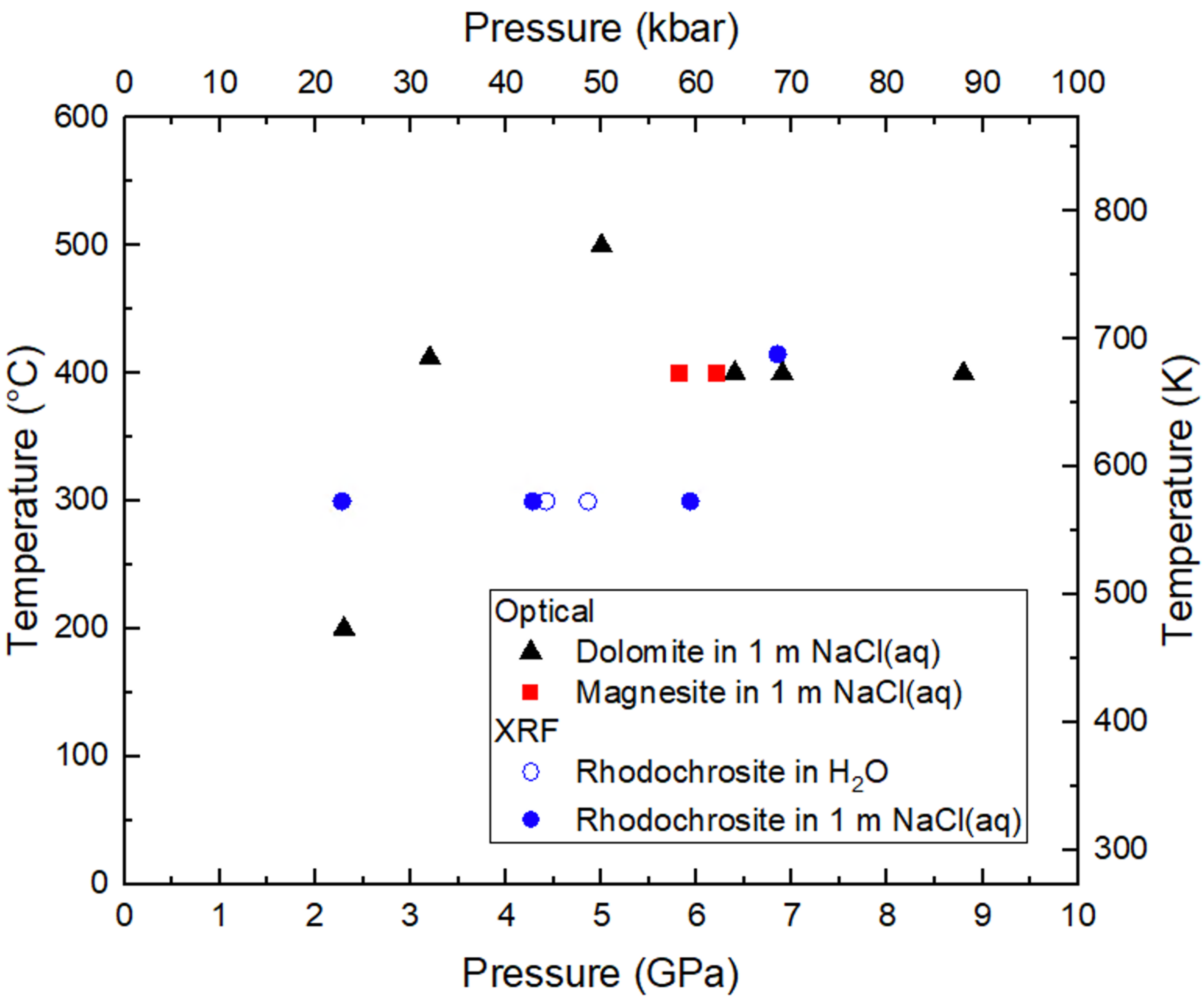

Supplementary Figure 2. Pressure and temperature conditions of the optical solubility and synchrotron XRF spectroscopy measurements.

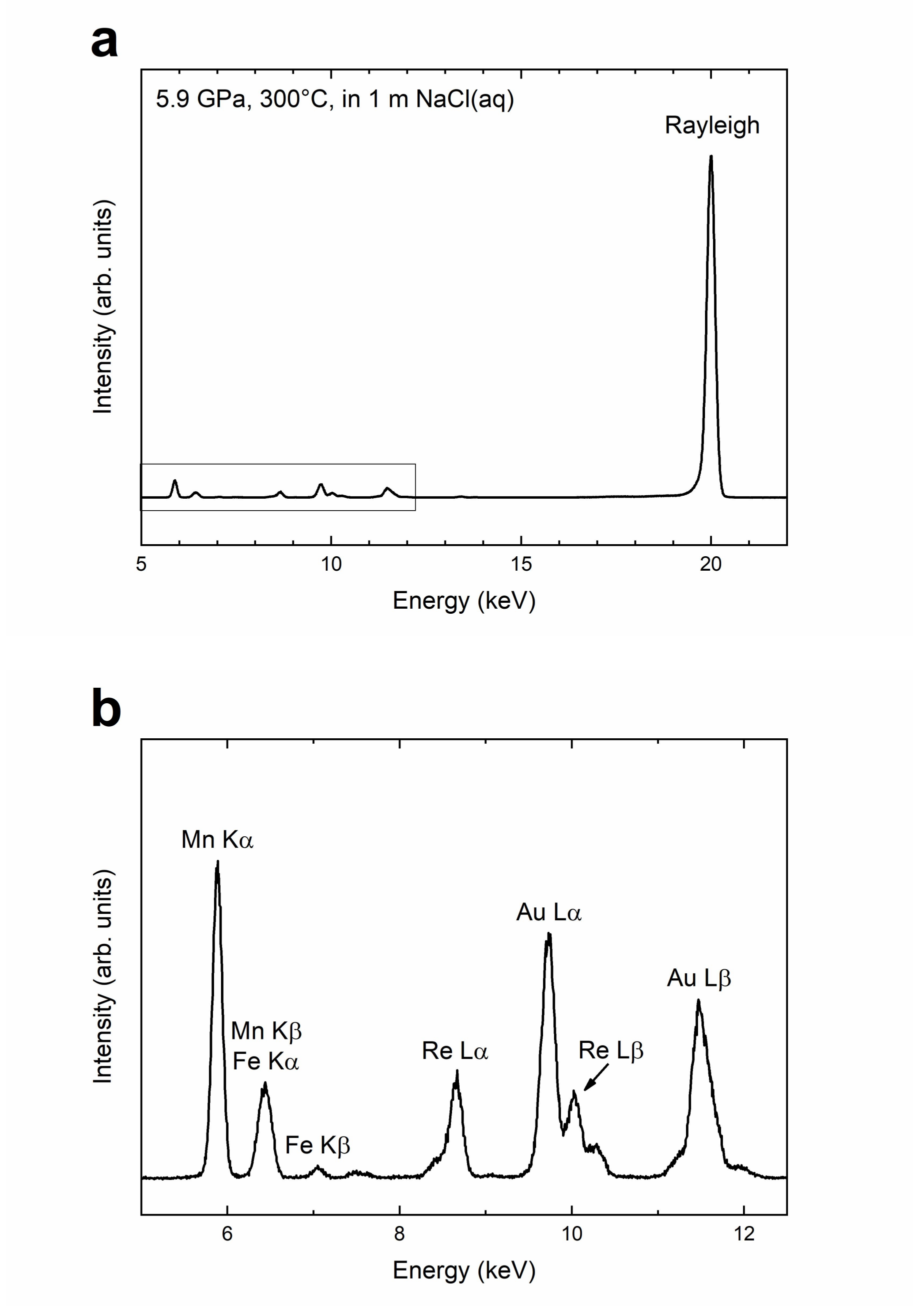

Supplementary Figure 3. An example of a raw synchrotron XRF spectrum of the fluid in equilibrium with rhodochrosite. The area defined by the rectangle in a) is enlarged in b). The Mn and Fe signal arises from the dissolution of rhodochrosite, while the Re and Au peaks originate from the dissolution of the Re gasket and its Au lining.
