# Supplementary material for: Deep carbon cycle constrained by carbonate solubility
Source: Nat Commun. 2021 Jul 14;12:4311. doi: 10.1038/s41467-021-24533-7 (PMC8280166; doi:10.1038/s41467-021-24533-7)
Supplement: Supplementary file 2 — Description of Additional Supplementary Files [file 41467_2021_24533_MOESM2_ESM.pdf]

Description of Additional Supplementary Files

File name: Supplementary Data 1:
Description: Compilation of subducting slab specific water and carbon fluxes

File name: Supplementary Data 2:
Description: Subducting slab specific carbon recycling

File name: Supplementary Data 3:
Description: Comparison of carbon recycling efficiency with CO_2 outputs from strong emitter volcanoes
